# Circulating MicroRNAs as Prognostic and Therapeutic Biomarkers in Breast Cancer Molecular Subtypes

**DOI:** 10.3390/jpm10030098

**Published:** 2020-08-22

**Authors:** Veronica Zelli, Chiara Compagnoni, Roberta Capelli, Katia Cannita, Tina Sidoni, Corrado Ficorella, Carlo Capalbo, Francesca Zazzeroni, Alessandra Tessitore, Edoardo Alesse

**Affiliations:** 1 Department of Biotechnological and Applied Clinical Sciences, University of L’Aquila, Via Vetoio, Coppito 2, 67100 L’Aquila, Italy; veronica.zelli@univaq.it (V.Z.); chiara.compagnoni@graduate.univaq.it (C.C.); roberta.capelli@graduate.univaq.it (R.C.); francesca.zazzeroni@univaq.it (F.Z.); edoardo.alesse@univaq.it (E.A.); 2 Center for Molecular Diagnostics and Advanced Therapies, University of L’Aquila, Via Petrini, 67100 L’Aquila, Italy; 3 Medical Oncology Unit, St Salvatore Hospital, Via L. Natali, 1, 67100, L’Aquila, Italy; kcannita@gmail.com (K.C.); tina_sidoni@yahoo.it (T.S.); corrado.ficorella@univaq.it (C.F.); 4 Department of Molecular Medicine, University of Rome “La Sapienza”, Viale Regina Elena 324, 00161 Rome, Italy; carlo.capalbo@uniroma1.it

**Keywords:** breast cancer, molecular subtypes, microRNA, circulating microRNA, biomarkers

## Abstract

Breast cancer (BC) is a common and heterogeneous disease, of which six molecular subtypes, characterized by different biological features and clinical outcomes, were described. The identification of additional biomarkers able to further connote and distinguish the different BC subtypes is essential to improve the diagnostic, prognostic and therapeutic strategies in BC patients. MicroRNAs (miRNAs) are short non-coding RNA involved in several physiological and pathological processes, including cancer development and progression. In particular, circulating miRNAs, which can be found in an adequately stable structure in serum/plasma of cancer patients, are emerging as very promising non-invasive biomarkers. Several studies have analyzed the potential role of circulating miRNAs as prognostic and therapeutic markers in BC. In the present review we describe circulating miRNAs, identified as putative biomarker in BC, with special reference to different BC molecular subtypes.

## 1. Introduction

Breast cancer (BC) is the most common cancer in women and the leading cause of cancer-related death [1], representing a serious concern for public health. At the molecular and clinical level, BC comprises heterogeneous forms, and the advancement of knowledge in the field showed that accurate molecular classification is necessary to significantly improve prognosis and treatment choice [2]. To date, six major BC molecular subtypes (Luminal A, Luminal B, HER2 positive, basal-like (triple negative), normal-like and claudin-low), characterized by different molecular features and clinical outcomes, were identified [3].

MicroRNAs (miRNAs) are short, single-stranded non-coding RNAs that regulate gene expression at the post-transcriptional level, the dysregulated expression of which is involved in some high-impact diseases, such as cancer [4,5]. In the last few years, great attention was addressed to the role of miRNAs as potential biomarkers in cancer: in particular, circulating miRNAs can be released from the tumor microenvironment and poured into the bloodstream, where they are stable and resistant to endogenous RNase activity [6], thus reflecting the homeostatic response of the organism to disease development and progression. For this reason, they are considered potential diagnostic, prognostic and therapeutic non-invasive biomarkers [7].

BC epigenetic mechanisms are not completely elucidated, but it is now undeniable that miRNAs play an important role in BC pathogenesis and progression, being able to specifically fine-tune different types of key genes with oncogenic, tumor suppressive properties, or also acting in DNA repairing mechanisms [8]. An increasing number of studies have focused on shedding light on the correlation between the complex biology of miRNA expression and BC features, with reference to the different types, clinical presentation, behavior, heterogeneous structure of tissues and gene expression profiles. Many studies have described the significant differential expression levels of groups of miRNAs with potential diagnostic/prognostic value in tissues or sera from BC patients—among them, several were also described as putatively playing a role in predicting BC therapy response [2,9,10]. 

The aim of this review is to provide an overview of the role of circulating miRNAs as prognostic and therapeutic biomarkers in BC, with focus on the different BC molecular subtypes.

## 2. Breast Cancer

BC is the most diagnosed cancer in women worldwide, accounting for about 11.6% of all cancer cases and 6.6% of all cancer-related deaths [1]. The annual incidence of BC is estimated at more than 2 million new cases, with the highest rates observed in Australia/New Zealand, Europe, and Northern America [1].

The most classical criteria for BC clinical management are tumor size, lymph-node status, presence of distant metastasis, expression of estrogen and progesterone receptors (ER, PR), expression level of human epithelial growth factor receptor 2 (HER2) [11,12]. In the last few decades, technical advancement focused on gene expression analysis made breast tumor classification possible, based on their own molecular features, leading to the generation of six major subgroups, characterized by differences in aggressiveness: Luminal A, Luminal B, HER2 positive, TN (triple negative, or basal-like), normal-like and, more recently, claudin-low [13,14,15,16]. 

The luminal subtype, mainly dominated by hormone receptor positive (HR+) tumors, and generally ER+, PR+, HER2 negative (HER2-), is the most common BC profile, accounting for about 60% of all BC tumors [17]. Compared to Luminal A, showing the most favorable prognosis, the Luminal B subgroup is characterized by higher recurrence risk, due to the expression of genes involved in cell proliferation (Ki67/MIB1). Depending on HER2 expression, Luminal B tumors can be further split into Luminal B/HER2+ and Luminal B/HER2- [3,12].

TN and HER2+ subtypes are characterized by HR- enriched tumors; in particular, the TN subtype lacks ER, PR, HER2 expression and shows cytokeratin (CK5/6, CK14) expression increase, whereas the HER2+ subgroup is dominated by tumors with a high expression of HER2 and HR loss [3,12,17]. Due to the above-mentioned molecular features, HER2+ tumors can benefit from anti-HER2 targeted therapy (trastuzumab, lapatinib), whereas TNBCs show the worst prognosis and are currently not eligible for any biological treatment.

**Figure 1:**
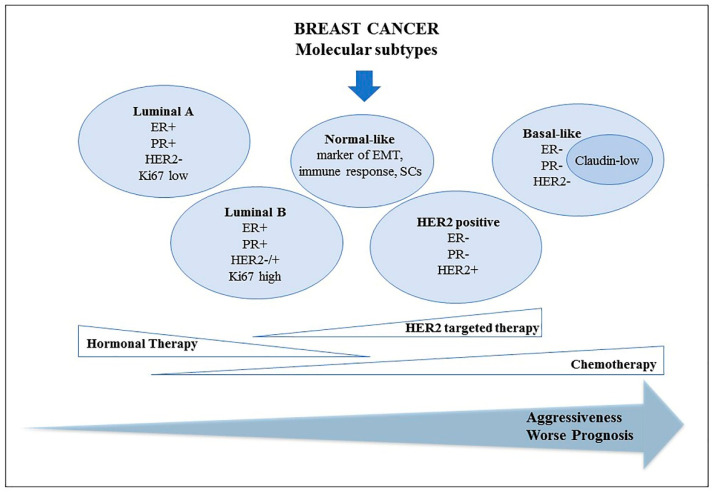
Classification of breast cancer molecular subtypes. Abbreviations: ER+, estrogen receptor positive; PR+, progesterone receptor positive; HER2+, human epithelial growth factor receptor 2 positive; EMT, epithelial-to-mesenchymal transition; SCs, stem cells.

Furthermore, in the normal-like subtype, a gene expression pattern similar to normal breast tissue was observed [3], whereas the claudin-low type is a less common and aggressive BC, mainly characterized by TN tumors with epithelial-to-mesenchymal transition features, immune response and cancer stem cell-like markers [16]. Overall, the advancement of knowledge of molecular mechanisms at the base of BC, leading to BC molecular classification, made the development and adoption of specific drugs for personalized medicine possible, greatly improving prognosis and BC tailored treatment (Figure 1).

Differences related to mutational landscape as well as a combination of gene expression patterns and copy number alterations were also observed among different BC cases, identifying subgroups of tumors characterized by similarities in somatic alterations [18,19,20], some of which were specific for the different molecular subtypes, described above [19,21,22]. Molecular profiling tools, such as Oncotype DX, MammaPrint, Prosigna, Endopredicit and Breast Cancer Index, are in use to predict BC prognosis and therapy response. In this regard, validation data of three extensive randomized prospective studies to select ER+/HER2- patients who could benefit from adjuvant chemotherapy plus endocrine therapy are available: two of them (TAILORx, RxPONDER) used Oncotype DX (21 relevant genes by qRT-PCR) which classified ER+ cancers in three groups based on “recurrence scores” [23,24]; the third study (MINDACT) used MammaPrint (70 genes by microarray) to define BCs with high and low recurrence risk [25,26]. However, more light can be shed on factors able to better refine BC molecular subtypes and identify novel non-invasive biomarkers with diagnostic, prognostic and predictive value. In this context, microRNAs are considered among the most promising molecules.

## 3. Circulating MiRNAs: From Tumor to Bloodstream

MiRNAs are short (18–22 nucleotides), single-stranded non-coding RNAs involved in the post-transcriptional regulation of specific target genes [27,28]. MiRNAs play a crucial role in several biological processes, such as cell cycle regulation and differentiation, apoptosis, metabolism and cell signaling [29,30,31]. On the other hand, the alteration of their expression may significantly contribute to the pathogenesis of many relevant diseases, including the development and progression of cancer. Depending on the role of their own target genes, miRNAs with oncogenic (oncomiR) or tumor suppressive (tumor suppressor miR) properties have been described at the tissue level [4]. In addition, the dysregulated miRNAs produced in the tumor microenvironment can be released into the bloodstream because of passive (apoptosis or necrosis of cancer cells) or active (secretion of exosomes and microvesicles) mechanisms [32].

Extracellular vesicles are involved in the process of cell communication by transporting different types of molecules, such as proteins, mRNA and microRNA, and can act both in the tissues in which they originate and in distant sites [33]. 

**Figure 2:**
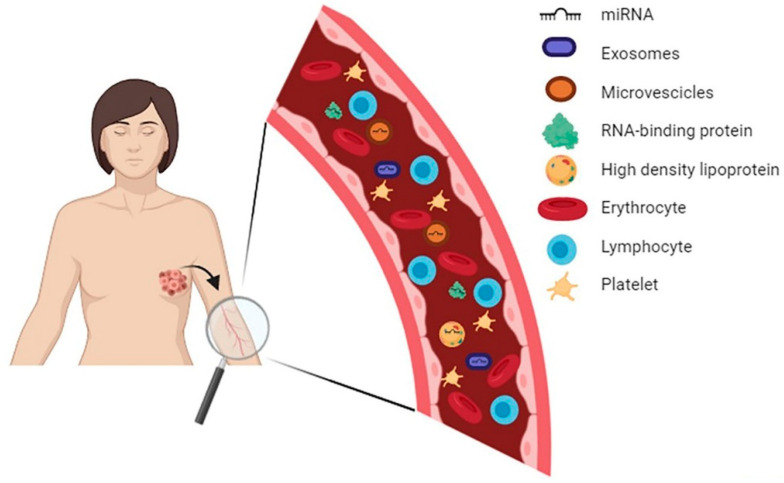
Schematic representation of miRNA release from tumor to bloodstream and compartmentalization of circulating miRNAs.

The inclusion of miRNAs within apoptotic bodies, exosomes and microvesicles, as well as the association of secreted miRNAs with RNA-binding proteins (Argonaute 2) or complexes of lipoproteins (HDL) [34], make circulating miRNAs very stable and RNase-protected, even in difficult conditions (e.g., low/high pH environment) [35] (Figure 2).

MiRNAs can be detected in plasma or serum via liquid biopsy by using specific and highly sensitive methods, such as real time quantitative reverse transcription-PCR [6,36], although the standardization of purification and analysis processes should be further improved [7,37]. 

Therefore, microRNAs produced by the tumor microenvironment, reflecting aberrant pathological processes, can pour into the bloodstream from which, due to the structure and nature that makes them resistant to RNase degradation, they can be easily recovered and analyzed, providing a source of characterizable and putatively suitable non-invasive disease biomarkers. 

## 4. Tissue MiRNAs in Breast Cancer Molecular Subtypes

In the last decade, thanks to the advancement of knowledge on miRNAs in cancer pathogenesis and progression, an extensive amount of research has been focused on the identification and characterization of altered miRNAs’ levels in BC. Several studies analyzed miRNA expression in BC tissues stratified in accordance with pathological features or subtypes. The dysregulation of different miRNAs was reported among the various BC molecular subtypes and their alteration was found to contribute to cancer progression and treatment response [38,39]. Furthermore, peculiar miRNA signatures, able to distinguish and differentiate BC subtypes, were identified [39]. For example, among the subtype-specific miRNAs, let-7c, let-7f and miR-10a were associated with Luminal A tumors; miR-18a, miR-135b, miR-93 and miR-155 were correlated with TNBC tumors; while miR-142-3p and miR-150 were associated with HER2+ tumors [40]. Moreover, the up-regulation of miR-342 was specifically observed in Luminal B cases [41].

Recently, Denkiewicz et al. [42] used bioinformatics and artificial intelligence to describe a wide BC molecular landscape. They integrated TCGA (The Cancer Genome Atlas) miRNA NGS dataset with clinical data to perform survival analysis, identify specific miRNAs for each BC subtype and analyze target genes and transcription factors targeting miRNAs as well. Among the 100 top microRNAs selected for each molecular subtype, they identified 44 miRNAs common to Luminal A, B, HER2-enriched and TN subgroups, and 12, 14, 9 and 15 miRNAs which were specific for each subtype. A set of 8 miRNAs with expression increases in stage II compared to I, was identified as well, with miR-10b-5p contributing to BC metastasis. The network analysis of miRNAs, target genes and transcription factors highlighted interesting interactions among them and relevant circuitries containing cancer key genes, such as TP53, BRCA1, Myc, ERS1, PI3K and pathways (e.g., KEGG breast cancer, apoptosis, p53, PI3K-Akt, MAPK, cell cycle). 

Other researchers [43] performed small RNA sequencing of a set of 186 tumor samples subdivided by ER and HER2 expression arrangement (ER+/HER+, ER-/HER+, ER+/HER-, ER-/HER-). They described that miRNA expression levels can be facilitated to discriminate specific subtypes—miR-4728 specifically identified HER2-enriched tumors and miR99a/let-7c/miR-125b differentiated Luminal A from Luminal B subgroup.

Nama et al. [44] performed a study on the worst prognosis TN subtype and identified miR-138 hyperexpression with diagnostic and prognostic value. MiR-138 led cells to proliferation, inhibited apoptosis in vivo, and the tumor suppressor candidate TUSC2 was shown to be a direct target of miR-138. Amorim et al. [45] investigated miRNAs expression levels in 139 Luminal BC and identified a panel of four miRNAs (miR-30c-5p, 30b-5p, 182-5p, 200b-3p) as independent predictors of endocrine therapy benefits. MiR-182-5p and 200b-3p provided prognostic information about recurrence following endocrine therapy.

MiR-193a-3p downregulation was observed in HER2+ BC cells and tissues, and Tang et al. [46] demonstrated that miR-193a-3p methylation led to the progression of HER2+BC by targeting GRB7, that was frequently co-expressed with HER2 and correlated with a metastatic phonotype, ERK1/2, the phosphorylation of which was increased by GRB7 and FOXM1, a transcriptional regulator overexpressed in HER2+ cancers and activated by cyclin–cyclin dependent kinase and ERK-induced phosphorylation. 

Overall, increasing evidence highlights the pivotal role and contribution of miRNAs in the progression and therapy response of different BC molecular subtypes and how the characterization of miRNA expression profiling could improve our understanding of BC heterogeneity.

## 5. Circulating MiRNAs in Breast Cancer Molecular Subtypes

Many studies analyzed circulating miRNAs with respect to BC molecular subtypes. Many of the microRNAs here described were already known to be dysregulated in cancer tissues and to play a role in tumor development and progression. Overall, cancer patients with different molecular features were compared in various ways, by often taking into consideration heterogeneous criteria for comparisons (e.g., HER2+ vs. HER2-; HER2+ vs. TNBC; Luminal vs. TNBC; Luminal vs. healthy controls; pre-therapy vs. post-therapy). The most recent literature is reported below and summarized in Table 1 to provide a clearer synopsis. 

### 5.1. Luminal Subtype

Several studies focused on analyzing significant differentially expressed miRNAs in sera from Luminal BC patients vs. healthy controls, or from paired pre- vs. post-therapy samples, providing helpful information to be used for diagnosis and prognosis evaluation.

McDermott et al. [47] took advantage of innovative in silico methodologies to select miRNAs of potential interest and prospectively analyzed blood RNAs of 54 patients with Luminal-like A BC, compared to 56 controls. Data were analyzed by an artificial neural network and the levels of differentially expressed miRNAs were further validated by qRT-PCR. Four miRNAs (miR-29a, -181a, -223, -652) showed expression decrease in patients, and binary logistic regression corroborated that three of them (miR-29a, -181a, -652) may differentiate between affected women and healthy controls, providing elements for specific Luminal A tumor subtype detection. 

Pre- and post-adjuvant chemotherapy serum levels of miR-21, already described as highly expressed in BC, and miR-145, acting as a tumor suppressor and down-regulated in BC, were examined in 52 Luminal A BC patients [48]. The authors did not evidence any statistical difference, even though both miRNAs were described as dysregulated in several BC subtypes in other studies, reported below. 

Cecener et al. [49] first analyzed miR-195 expression in 96 Luminal A and B BC tissues and then in pre- and post-operative blood samples from Turkish patients. They described miR-195 downregulation in BC with respect to normal adjacent tissues and significant down-regulation in post- vs. pre-operative samples. Moreover, a significant miR-195 blood increase was detected in Luminal B pre-operative specimens, suggesting that it might be considered as an early biomarker.

Pre- and post-surgery and treatment (chemo/radiotherapy) serum levels of the oncomiRs miR-21, miR-155, miR-10b, and the tumor suppressor Let-7a were analyzed in 30 Luminal A BC patients with respect to 10 unaffected controls [50]. MiR-155, -21 and -10b expression showed significant increase in BC; on the contrary, let-7a level decrease was detected in comparison to controls. Furthermore, serum levels of miR-21, -155, -10b decreased, whereas those of let-7a increased after surgery and therapy. The most significant differences were identified for miR-155. Overall, the results showed that Luminal A BC treatment led to a decrease in oncomiRs expression and an increase in contextual tumor suppressor miR, providing clinical information to be possibly used for monitoring the trend of the disease. 

Another study [51] analyzed miRNA profiling in plasma specimens from age-matched patients with locally-confined or metastatic Luminal A BC and found significant higher miR-331 levels in metastatic patients with respect to local Luminal A and healthy controls. Conversely, miR-195 appeared hypo-expressed in metastatic patients and, used in combination to miR-331 levels, showed the ability to distinguish metastatic from Luminal A cancers.

### 5.2. HER2 Positive Subtype

The role of significant differentially expressed circulating miRNAs in HER2+ molecular subtypes was investigated in several studies to evaluate their potential to distinguish BCs based on HER2 expression and to provide prognostic information. 

MiR-21, -10b, -19a, all linked to BC cell proliferation, invasion and metastasis, were prospectively analyzed in a cohort of 113 BCs for examining their putative use as biomarkers for more aggressive disease [52]. MiR-21 increased levels were observed for non-metastatic HER2+ with respect to HER2- patients, whereas higher miR-10b expression was detected in metastatic HER2+ cancers compared to HER2-. Patients with metastatic inflammatory BC showed miR-19 level increase compared to metastatic non-inflammatory BC. The same miR was correlated with longer PFS (progression free survival) and OS (overall survival) in patients with metastatic HER2+ inflammatory BC, indicating that miR-19 can predict favorable prognosis for this specific BC subtype.

In a case–case study [53], the association between serum miRNA expression and cancers stratified for ER, PR, HER2 expression and lymph-node status was analyzed. Seven significant differentially expressed miRNAs (miR-183, -660, 29a, -93, -378, -4281, -4283) were identified in HER2+ vs. HER2- cases, and 10 (miR-320b, -320d, -145, -320e, -1307, -767-3p, -125b, -605, -1825, -124) in lymph-node positive vs. negative patients, providing molecular markers associated to tumors with different features.

Hamdi et al. [54] analyzed circulating miRNA expression in inflammatory and non-inflammatory BC and described lower miR-15a serum levels in the HER2+ subtype, offering a distinctive element for such a BC subgroup. 

### 5.3. TNBC Subtype

TNBC corresponds to the worst prognosis BC subtype: for this reason, there is an urgent need to identify diagnostic, prognostic and predictive biomarkers. In this context, significant differentially expressed circulating miRNAs can be considered suitable and promising biomarkers.

Salhberg et al. [55] identified in sera from 60 primary TNBCs, and further validated in an additional independent cohort of 70 TNBCs, a panel of four miRNAs (miR-18b, -103, -107, and -652), as a predictor of tumor relapse and OS. The signature was highly expressed in the relapsing group, allowing patients’ stratification and potentially guiding the choice of the most appropriate therapy.

The role of ADAM8, a protein known to be correlated with invasive and metastatic features in TNBC, was investigated in both in vitro and in vivo models by Das et al. [56]. ADAM8 was able to induce miR-720 expression by activating the ERK signaling cascade, and serum levels of miR-720 were found significantly higher in TNBC patients highly expressing ADAM8. 

Another study showed that decreased serum levels of miR-940, a microRNA previously investigated in several types of cancer, were detected in BC patients with respect to controls and predicted worst prognosis in TNBC [57].

Finally, the role of the miR-34a, a tumor suppressor microRNA already described in different cancers, was examined by Li et al. [58] in TNBC patients compared with healthy controls. The authors detected miR-34a downregulation in patients, further demonstrating that miR-34a inhibition in TNBC cells enhanced tumor proliferation and glucose uptake by upregulating the glucose uptake and transporter 1 (GLUT1) gene, which plays a key role in the transport of glucose. 

### 5.4. Comparison among Different BC Subtypes

In order to shed light on the BC heterogeneity, several studies were based on the comparison among different BC subtypes with the aim to identify circulating biomarkers able to distinguish BC subtypes and aggressiveness.

Eichelser et al. [59] analyzed after-surgery and before-chemotherapy serum levels of six microRNAs known to be involved in tumor development and metastasis spread (miR-10b, -17, -34a, -93, -155, and -373) in 120 patients with primary tumor, equally distributed in ER+/PR+, HER2+ and TNBC groups, 32 patients with metastasis and 40 healthy controls. MiR-34a, -93 and -373 were significantly higher in the primary BC group vs. controls, indicating their connection to cancer and potential early diagnostic value. In particular, for miR-373, an AUC (area under the curve) value of 0.879 (*p* = 0.0001), highlighting the relevant difference between the two groups considered, was reported. An increased miR-373 level was also observed in the HER2- patients, whereas different levels of miR-34a and miR-17 were shown by women with ER/PR negative and positive statuses, respectively. Moreover, miR-17 and miR-155 differentiated the primary BC group from the metastatic one, where decreased expression was observed. Altogether, the results suggested that such dysregulated miRNAs could be involved in the metastatic spread of BC subtypes. 

Furthermore, the same research group examined pre-surgery and -chemotherapy blood levels of cell-free miR-101, -372 and -373 in 168 BC patients, 19 of which had benign disease, and 28 healthy controls. The same miRNAs were also examined in exosomes obtained from 50 patients and 12 healthy controls belonging to the same cohort. The study highlighted significantly different levels of miR-101 and miR-373 between BCs and benign samples, the higher expression of circulating exosomal miR-373 in TN compared to Luminal cases and in ER-/PR- cancers, compared to HR+ as well. Furthermore, miR-373 overexpression was able to induce ER down-regulation and apoptosis inhibition in the in vitro cell models, highlighting that miR-373 was correlated to a more aggressive BC phenotype [60]. 

Eighty-five paired tumor samples and sera were compared to healthy subjects and 15 benign fibroadenomas by Thakur et al. [61]. The significant hyper-expression of oncomiRs-21, -221, -210, and the hypo-expression of tumor suppressors miR-195 and -145, were observed in TN with respect to triple positive BCs, indicating a possible correlation with the aggressiveness of the disease. Furthermore, despite its tumor-suppressive role, let-7a was found up-regulated in TNBCs. 

Huo et al. [62] identified a highly reliable miRNAs prognostic signature of seven miRNAs to distinguish BC patients with and without recurrence. Among them, miR-21-5p, -375, -205-5p, -194-5p appeared significantly up-regulated, whereas miR-382-5p, -376c-3p, -411-5p were down-regulated in recurrent patients. This signature was suitable for both HR+ and TNBCs.

A panel of 18 distinct up-regulated circulating miRNAs in 23 BC individual patients with respect to nine healthy controls was identified by Hamam et al. [63]. Nine of those (miR-4270, -1225-5p, -188-5p, -1202, -4281, -1207-5p, -642b-3p, -1290 and -3141) were subsequently validated on 46 patients and 14 controls. The results demonstrated higher miRNA levels in stages I–III compared to stage IV, providing a putative distinctive element to be used for early diagnosis. Moreover, higher expression levels were detected in HER2+ and TN with respect to Luminal subtypes. Interestingly, this panel did not include some of the miRNAs most frequently hyper-expressed in breast tissues, leading us to hypothesize that circulating miRNAs could reflect not only the tumor microenvironment, but also the global homeostatic response to the disease. 

Qattan et al. [64] analyzed the microRNA expression profiles in plasma from 36 patients with TNBC, 57 with Luminal BC, and from 34 disease-free controls. Data were also matched with RNAseq TCGA datasets. A panel of 18 miRNAs was isolated and 10 unique miRNAs were identified. Among them, let-7 and miR-195 were up-regulated in Luminal BCs and TNBCs, respectively. Conversely, data from TCGA showed a very low expression of both miRNAs in cancer tissues and high expression in adjacent non-cancer ones, probably indicating that tumor cells might selectively export miRNAs throughout the oncogenesis process. 

The expression levels of circulating miR-16, miR-21 and miR-155, listed among the most hyper-expressed microRNAs in BC, and miR-195, considered as a BC-specific miRNA, were analyzed by Fan et al. [65] in 49 women with BC and 19 healthy controls with no history of BC. Serum levels of all four miRNAs were significantly increased in stage I BCs and in HER2-overexpressing, Luminal A/B and TN subtypes compared to controls, as further confirmed by ROC curve analysis, where high AUC values were obtained (>0.8), suggesting a possible use as biomarkers for early diagnosis.

Niedźwiecki et al. [66] compared the serum expression of oncomiRs miR-21, miR-10b and of miR-200c, the downregulation of which was described as cell migration and metastasis promoter, in 46 TN and ER+/PR+ BC patients. They found significantly lower miR-200c amounts in the TNBC group, highlighting the possible role of miR-200c levels as a biomarker predictor of worst prognosis and metastasis. Conversely, no significant differences were detected for miR-10b and -21.

In a case-control study, Souza et al [67] identified a panel of 42 significant differentially expressed miRNAs in 54 (Luminal A, Luminal B, Luminal B HER2+, HER2-, TN) early stage BC patients—among them, 19, 8, 10, 4 of were significantly differentially expressed in Luminal A, Luminal B, Luminal B-HER2+, HER2 enriched types, respectively. Regarding TNBC, just miR-25-3p showed significant up-regulation and was able to distinguish the TN patients from heathy controls. Overall, the study demonstrated the possibility to obtain non-invasive molecular signatures for BC molecular subtypes classification.

The serum levels of miR-21, often overexpressed in BC, and miR-206, playing a role in the ERα regulatory loop, were examined in 75 BC patients stratified on ER, PR and HER2 statuses [68]. MiR-206 was found hyper-expressed in Luminal A and B subtypes, whereas an miR-21 increase was observed in HER2+ and TNBCs. Furthermore, the expression of the latter microRNA was negatively associated with ER and PR expression level, and positively with HER2 and malignancy. Conversely, an opposite behavior was described for miR-206. Therefore, miR-21 and -206 correlated with HR and HER2 expression and could distinguish Luminal from the worst prognosis HER2+/TNBC subtypes.

Finally, Ozawa et al. [69] analyzed miRNAs extracted from extracellular vesicles of 16 Luminal A and 15 TNBC patients compared to the controls. They selected four miRNAs (miR-142-5p, -150-5p, -320a, -4433b-5p) and demonstrated that miR-142-5p, miR-320a, and miR-4433b-5p were able to distinguish BC patients from healthy individuals with 93% sensitivity and 68% specificity. Furthermore, the association of miR-142-5p and miR-320a discriminated Luminal A BC from the control with 100% sensitivity and 93% specificity, whereas decreased levels of miR-142-5p and miR-150-5p were correlated with grade III tumors and the lower expression of miR-142-5p and miR-320a with higher tumor size.

Overall, the above-reported results, arising from studies based on the analysis and comparison of breast cancers subtypes, often differentially stratified and arranged, clearly demonstrate that circulating miRNAs levels can provide interesting data to better define the biological heterogeneity of BC and support diagnostic, prognostic and predictive clinical evaluation. 

## 6. BC Molecular Subtypes: Differentially Expressed MiRNAs in Response to Therapies

It is known that different BC subtypes can differently respond to therapies. For example, the Luminal A subtype is responsive to endocrine therapy—conversely, it is less sensitive to chemotherapy. Furthermore, thanks to the generation of targeted drugs, over the last few decades, trastuzumab and lapatinib have greatly improved prognosis of HER2+ BCs. However, a percentage of initially responding patients can later develop resistance to therapeutic treatments. Several studies were aimed at investigating the significance of differentially expressed circulating miRNAs in response to therapies (Table 2).

A cohort of 68 Luminal A patients subjected to neoadjuvant epirubicin plus paclitaxel chemotherapy was analyzed by Li et al. [70]. Among eight serum differentially expressed miRNAs, they described miR-19a and miR-205, which were hyper-expressed in the resistant group, as significantly able to predict the sensitivity of Luminal A BCs to this therapeutic regimen, providing non-invasive biomarkers for better defining in advance the expected response. 

Circulating levels of miR-21, -210 and -373 were assayed in 127 HER2+ patients pre- and post-neoadjuvant therapy (chemotherapy combined to trastuzumab/lapatinib) [71]. Significantly higher miR-21, -210 and -373 levels were detected before and after therapy in BC patients compared to healthy controls. MiR-21, -210 and -373 increase was further observed in patients after therapy. The expression of miR-21 before and after therapy was correlated with OS, regardless of the type of anti-HER2 treatment, and higher circulating miR-373 expression was observed in advanced stage BC, highlighting the potential role of those microRNAs in prognosis assessment. On the contrary, the association between serum microRNA and pathological response was not evidenced.

In a recent article, Li et al [72] identified a panel of 13 serum miRNAs (miR-720, -4716-5p, -17-3p, -451a, -16-5p, -451b, -940, -10b-3p, -30b-3p, -4310, -494, -22-3p, -29a-5p) differentially expressed in metastatic HER2+ women who exhibited distinct responses to trastuzumab. Among them, four microRNAs (miR-940, -451a, -16-5p, -17-3p), specifically targeting molecules involved in pathway conferring anti-HER2 resistance of BC cells (SRC, PTEN, IGFR1), were used to predict sensitivity to trastuzumab.

The oncomiR 21 was identified as a prognostic and predictive factor for the response to trastuzumab in 20 HER2+ metastatic BCs [73]. MiR-21 level decrease, also correlated to time to progression, was observed after trastuzumab treatment, with higher difference in responders. 

Rodríguez-Martínez et al. [74] prospectively analyzed 53 women with localized BC who underwent neoadjuvant chemotherapy. Afterwards, among them, six developed metastatic disease. The authors reported pre-therapy serum exosomal miRNA-21 and -105 increases in metastatic patients with respect to non-metastatic women and controls. Moreover, higher miR-222 expression levels were detected in basal-like and in Luminal B vs. A tumors and correlated to progesterone receptor status and Ki67. Under treatment, the levels of miR-21 were correlated to tumor size and, in an inverse manner, to Ki67. Global miRNA-21, -222, -155 high levels were significantly linked to circulating cancer cells into the blood.

Microarray analysis was performed to discriminate trastuzumab sensitive and resistant cell lines and isolate differentially-expressed miRNAs [75]. Among the most interesting, miR-200b, -135b, -29a, -224 were validated and showed differential expression on sera from HER2+ patients (200b, 135b, 29a up-regulated; 224 down-regulated in trastuzumab-resistant patients), demonstrating a correlation with response to therapy.

Liu et al. [76] examined the relationship between serum miRNAs and the aresponse of HER2+ patients treated with neoadjuvant chemotherapy plus trastuzumab. MiR-21 decreased levels were observed throughout treatment administration, demonstrating a significant correlation with prognosis and clinical response.

Circulating miRNAs as putative biomarkers of the response to neoadjuvant therapy of TNBCs were examined by Ritter et al. [77]. After analyzing the expression levels of miR-7, -9, -15a, -17, -18a, -19b, -21, -30b, -222 and -320c in triple positive and TNBC cell models treated with several chemotherapeutic drugs, they described a putative association between miR-17, -19b and -30b expression serum levels and neoadjuvant chemotherapy-driven complete clinical response, highlighting the importance of these miRNAs in monitoring therapeutic outcome. The decreased expressions of miR-21 and miR-195 were observed in patients who responded to neoadjuvant chemotherapy with respect to non-responders. MiR-21 was described as a treatment predictor and, associated with miR-145, was found significantly reduced in responder Luminal cases [78].

## 7. Conclusions

MicroRNAs have emerged as key regulators of BC pathogenesis, progression and treatment response. Recently, some studies have focused on analyzing dysregulated BC miRNAs in plasma/serum to identify novel groups of biomarkers for predicting prognosis and, more efficiently, therapy response, also with reference to specific BC subtypes.

Several circulating miRNAs were described as differentially expressed in BC molecular subgroups, providing elements to be used not only to examine the biological heterogeneity of breast tumors in more depth, but also to stratify patients and provide a decisional support for clinical management. To date, the availability of sensitive and high-performance technologies, such as qRT-PCR or small-RNA NGS sequencing, offers great opportunity to specifically and sensitively assess circulating miRNAs’ expression levels, although the complexity of serum/plasma specimens still requires the improvement and standardization of analytical procedures to ameliorate the generation of data suitable for clinical utility. In addition, more extensive serum/plasma miRnome profiling could be beneficial and helpful to highlight significant differences for isolating groups of multiple and associated putative biomarkers to be further validated. Furthermore, to examine in depth mechanisms related to BC subtypes and expand the knowledge of the molecular landscape, it would be interesting and promising to characterize genes targeted by differentially-expressed miRNAs as well. 

The studies here reviewed were based on cohorts of patients often enrolled on the basis of different clinical/therapeutic features and, in certain situations, showed conflicting results. However, among the miRNAs here discussed, some deserve interest and could be subjected to deeper examination. For example, it would be relevant to further analyze miR-21, -10b, -378, -373, -188, the expressions of which seems to provide more coherent predictive and/or prognostic information, especially for HER2+ BCs. All the above-mentioned microRNAs were described in tumorigenesis. MiR-21 is one of the most well-studied microRNAs in cancer. It targets genes (e.g., PTEN, SPRY2, TIMP3, RECK) involved in the negative regulation of cell proliferation, apoptosis, metastasis and invasion pathways, such as PI3K/AKT, ERK/MAPK, and VEGFA. Furthermore, miR-21 is considered as a diagnostic, prognostic and predictive biomarker in several tumor types, including BC [79,80,81]. In the same way, miR-10b was described as an oncomiR in BC by targeting tumor suppressive genes (e.g., PDCD4, PTEN, TPM1), thus promoting proliferation, invasion and metastasis, and predicting the worst BC prognosis [82]. MiR-378 is known to suppress migration and invasion in BC cells and mouse models by targeting Runx1, one of the most mutated genes in BC [83], and Yin et al. [84] showed miR-378 up-regulation in BC patients vs. controls. MiR-373 was shown to enhance cell migration by suppressing ITGA2, the lack of which plays a relevant role in metastasis spread [85]; moreover, it was found hyper-expressed in TNBC [86]. Multiple roles were reported for miR-188, which negatively regulated cell proliferation and migration by targeting IL6ST [87] or enhanced apoptosis by suppressing the anti-apoptosis and pro-survival Rap2c [88].

In conclusion, further studies focused on the analysis of large, well-defined and stratified cohorts of patients are needed to validate the promising results already obtained and to identify novel miRNAs to be used as non-invasive prognostic and predictive biomarkers in BC subtypes. This will provide advantageous complementary knowledge not only about BC genetic arrangements and molecular features, thus shedding light on BC biological heterogeneity, but also on their use in the field of personalized medicine for optimizing diagnostic and prognostic clinical evaluation, as well as therapeutic options. 

## Figures and Tables

**Table 1:** Circulating microRNAs identified in the different breast cancer subtypes.

MicroRNAs	Expression Level/Prognostic Value	Reference
miR-29a, -181a, -223, -652	Decreased level in Luminal A cases compared to healthy controls	[47]
miR-195	Decreased level in post-surgery Luminal A and B cases. Significant increase in pre-operative Luminal B	[49]
miR-155, -21 and -10b	Higher level in Luminal A cases compared to controls	[50]
let-7a	Lower expression in Luminal A cases compared to controls	
miR-331	Higher level in metastatic with respect to local Luminal A cases and healthy controls	[51]
miR-195	Lower level in metastatic with respect to local Luminal A cases and healthy controls	
miR-21	Higher in non-metastatic HER2+ compared to HER2- cases	[52]
miR-10b	Higher in metastatic HER2+ compared to HER2- cases	
miR-19a	Favorable prognosis in patients with metastatic HER2+ inflammatory BC	
miR-183, -660, 29a, -93, -378, -4281	Down-regulated in HER2+ vs. HER2-	[53]
miR-4283	Up-regulated in HER2+ vs. HER2-	
miR-15a	Lower level in HER2+ vs. HER2- cases of inflammatory BC	[54]
miR-18b, -103, -107, -652	Predictors of tumor relapse and OS in TNBC (highly expressed in relapsing group)	[55]
miR-720	Higher level in TNBC cases with invasive and metastatic features	[56]
miR-940	Down-regulated and predictor of worst TNBC prognosis	[57]
miR-34a	Down-regulated in TNBC cases vs. healthy controls	[58]
miR-373	Up-regulated in HER2- vs. HER2+	[59]
miR-34a	Increased expression in HR- vs. HR+	
miR-17	Decreased expression in in HR+ vs. HR-	
miR-373	Higher in TNBC compared to Luminal BC and in HR- compared to HR+	[60]
miRs-21, -221, -210, let-7a	Higher expression in TNBC compared to triple positive cases	[61]
miR-195, -145	Lower in TNBC compared to triple positive cases	
miR-21-5p, -375, -205-5p, -194-5p	Up-regulated in HR+ and TNBC recurrent patients	[62]
miR-382-5p, -376c-3p, -411-5p	Down-regulated in HR+ and TNBC recurrent patients	
miR-4270, -1225-5p, -188-5p, -1202, -4281, -1207-5p, -642b-3p, -1290, -3141	Higher in HER2+ and TNBC with respect to Luminal cases	[63]
let-7a	Up-regulated in Luminal BC	[64]
miR-195	Up-regulated in TNBC	
miR-16, -21, -155, -195	Higher level in BC (regardless of the subtype) compared to controls	[65]
miR-200c	Lower expression in TNBC vs. ER+/PR+, predicts worst prognosis	[66]
mir-526b, -6503, -487b, -543, -627, -3614, -18b, -887, -30e, -132, -4647	Down-regulated in Luminal A cases vs. controls	[67]
mir-122, -376a, -196a, -301b, -23a, -3065, -548ah, -25, -335, -873	Up-regulated in Luminal A cases vs. controls	
mir-873, -188, -146a, -422a, -1283, -128-1	Down-regulated in Luminal B cases vs. controls	
mir-502, -548a, -548ar, -548ah, 203	Up-regulated in Luminal B cases vs. controls	
mir-221, -582, -1302, -449b, -888, -378, -299, -887	Down-regulated in Luminal B HER2+ cases vs. controls	
mir-25, -379, -933, -615	Up-regulated in Luminal B HER2+ cases vs. controls	
mir-584, -615, -1283	Down-regulated in HER2 enriched cases vs. controls	
mir-548ar	Up-regulated in HER2 enriched cases vs. controls	
miR-25-3p	Up-regulated in TNBC cases vs. controls	
miR-206	Up-regulated in Luminal A and B cases	[68]
miR-21	Up-regulated in HER2+ and TNBC	
miR-142-5p, -320a	Higher in Luminal A cases compared to controls	[69]

Abbreviations: +, positive; -, negative; BC, breast cancer; TNBC, triple negative breast cancer; OS, overall survival.

**Table 2:** Role of circulating microRNAs in response to therapies of breast cancer subtypes.

MicroRNAs	Expression Level/Prognostic Value	Reference
miR-19a, -205	Up-regulated in chemotherapy resistant compared to sensitive Luminal A cases	[70]
miR-21, -210, -373	Higher in HER2+ cases, before and after neoadjuvant therapy (chemotherapy combined to trastuzumab/lapatinib), compared to controls	[71]
miR-720, -4716-5p, -17-3p, -451a, -16-5p, -451b, -940, -10b-3p, -30b-3p, -4310, -494, -22-3p, -29a-5p	Differentially expressed in metastatic HER2+ cases in relation to trastuzumab response	[72]
miR-940, -451a, -16-5p, -17-3p	Predictors of trastuzumab sensitivity in metastatic HER2+ cases	
miR-21	Decreased level in HER+ cases after trastuzumab treatment: prognostic and predictive factor of trastuzumab response	[73]
miR-21, -105	Increased level in pre-neoadjuvant chemotherapy metastatic BC cases compared to non-metastatic cases and controls	[74]
miR-200b, -135b, -29a	Up-regulated in HER2+ trastuzumab-resistant cases	[75]
miR-224	Down-regulated in HER2+ trastuzumab-resistant cases	
miR-21	Predictor of therapy response (neoadjuvant chemotherapy plus trastuzumab) in HER2+ cases	[76]
miR-17, -19b, -30b	Predictors of response to neoadjuvant chemotherapy in TNBC cases	[77]
miR-21, -195. -145	Predictors of response to neoadjuvant chemotherapy in Luminal BC cases	[78]

Abbreviations: +, positive; -, negative; BC, breast cancer; TNBC, triple negative breast cancer.
